# Using the Infant Sibling-Design to Explore Associations Between Autism and ADHD Traits in Probands and Temperament in the Younger Siblings

**DOI:** 10.1007/s10803-023-06047-x

**Published:** 2023-06-24

**Authors:** Linn Andersson Konke, Terje Falck-Ytter, Emily J. H. Jones, Amy Goodwin, Karin Brocki

**Affiliations:** 1 Department of Psychology, Uppsala University, Box 1225, 751 42 Uppsala, Sweden; 2 Department of Women’s and Children’s Health, Karolinska Institutet Center of Neurodevelopmental Disorders (KIND), Centre for Psychiatry Research, Karolinska Institutet, Stockholm, Sweden; 3 Stockholm Health Care Services, Stockholm County Council, CAP Research Centre, Stockholm, Sweden; 4 Development and Neurodiversity Lab (DIVE), Department of Psychology, Uppsala University, Uppsala, Sweden; 5 The Swedish Collegium for Advanced Study (SCAS), Uppsala, Sweden; 6 Center for Brain and Cognitive Development, Birkbeck, University of London, London, UK; 7 Department of Forensic and Neurodevelopmental Sciences, Institute of Psychiatry, Psychology & Neuroscience, King’s College London, London, UK

**Keywords:** Temperament, Autism spectrum disorder, Attention-deficit/hyperactivity disorder, Etiology, Developmental substructure

## Abstract

The purpose of the current study was to use the infant sibling design to explore whether proband traits of autism and ADHD could provide information about their infant sibling’s temperament. This could help us to gain information about the extent to which infant temperament traits are differentially associated with autism and ADHD traits. We used parent-ratings of autistic traits and ADHD traits (CRS-3) in older siblings diagnosed with autism (age range 4 to 19 years), and their infant siblings’ temperament traits (IBQ) at 9 months of age in 216 sibling pairs from two sites (BASIS, UK, and EASE, Sweden) to examine associations across siblings. We found specific, but modest, associations across siblings after controlling for sex, age, developmental level and site. Proband autistic traits were specifically related to low levels of approach in the infant siblings, with infant developmental level explaining part of the variance in infant approach. Proband ADHD traits were specifically related to high levels of infant activity even after controlling for covariates. Our findings suggest that proband traits of autism and ADHD carry information for infant sibling’s temperament, indicating that inherited liability may influence early emerging behaviours in infant siblings. The impact of sex, age, developmental level and site are discussed.

Autism Spectrum Disorder (ASD; hereafter referred to as autism) is a heritable and heterogeneous neurodevelopmental condition characterized by qualitative differences in social communication and repetitive, restricted behaviors or interests (Lord et al., 2000; Rutter et al., 2003). There is a high co-occurrence between autism and Attention Deficit/ Hyperactivity Disorder (ADHD), but evidence of their shared heritability is currently mixed. While twin and family studies indicate shared genetic factors (Ghirardi et al., 2018; Rommelse et al., 2010; Simonoff et al., 2008; Tick et al., 2016), more recent genome-wide association studies suggest only modest genetic associations between autism and ADHD (Mattheisen et al., 2021). One possibility is that the overlap of autism and ADHD traits arise from a combination of aggregated inherited neurobehavioral susceptibilities that constitutes both specific and non-specific liabilities (Constantino et al., 2021). Once the characteristic features of autism emerge, the liabilities leading to these traits might have changed in character (Constantino, 2018; Constantino & Charman, 2016), which indicate a need for early identification of potential developmental markers.

There is an established literature on the development of infants with a family history of autism, with similar research efforts emerging in the ADHD field (Miller et al., 2023; Johnson et al., 2015). The prevalence estimates for autistic children with co-occurring ADHD traits range between 30 and 80% (Rommelse et al., 2010), and younger siblings with a family-history of autism are at elevated likelihood for both conditions (Miller et al., 2018a, 2018b). Thus, studies of infants with older siblings with autism, who have a broad range of clinical and phenotypic outcomes, are informative in the context of ADHD (Shephard et al., 2019). Given the infant sibling design, moving beyond categorical diagnosis to examine dimensional measures of proband traits may be an informative and cost-effective proxy of inherited genetic liability for autism. Phenotypic congruence between biological siblings with autism seem to be evident in domains other than autistic traits, such as language and adaptive behaviors (Goin-Kochel et al., 2008; MacLean et al., 1999). Recent work found that probands’ autism traits predicted diagnostic outcome in the younger siblings at 24 months and that traits such as adaptive behaviour and communication, were associated among proband-sibling pairs where both received a later autism diagnosis. Moreover, in a longitudinal MRI study by Girault et al. (2022), proband traits explained variation in brain phenotypes related to the visual circuitry in the infant siblings. Taken together, these studies suggest that applying a familial trait approach is a sensitive way of capturing inherited autism liability, with the possibility to reduce time to gather information about early developmental mechanisms related to autism and ADHD. Finally, key targets should be early markers that precedes the onset of autism and ADHD characteristics.

Temperament is defined as a biologically-based reactivity and regulation in behavior and emotion which develops in interaction with the environment (Shiner et al., 2012). Changes in temperament may contribute to liability for autism and ADHD (Goldsmith et al., 2005). Twin-studies indicate that the associations between autism, ADHD and alterations in temperament, in particular negative affect (Park et al., 2017), are partly due to genetic effects (Kerekes et al., 2013). This indicate overlap at both phenotypic and etiological levels across temperament variations and neurodevelopmental conditions (Martel et al., 2014; Tackett, 2006; Tackett et al., 2013). Studying aspects of temperament as part of the etiology of autism and ADHD is in line with the Research Domain Criteria (RDoC) approach (Insel et al., 2010). The RDoC focuses on mental health from a dimensional perspective with a specific emphasis on negative valence (e.g., fear and anxiety) and positive valence (i.e., approach) and sensorimotor systems (e.g., activity level) (Insel et al., 2010). Recent additional intents have been made to integrate developmental principles into the RDoC framework (Beauchaine & Hinshaw, 2020; Conradt et al., 2021; Garber & Bradshaw, 2020). Rothbart´s model of temperament (Rothbart, 1981) taps the RDoC domains well. In this model, reactivity refers to excitement and arousal of physiological responses to the environment and can be of positive (surgency) or negative (negative affect) valence, whereas regulation refers to the ability to modulate reactivity by means of attention shifting, self-soothing, or in interaction with a caregiver (Gartstein & Rothbart, 2003). Notably, variation in temperamental reactivity may serve as useful tools for explaining heterogeneity across (Visser et al., 2016) and within conditions (Garon et al., 2022; Hendry et al., 2020).

Differences in temperament have been linked to autism and ADHD using both categorical and dimensional approaches (Visser et al., 2016), with a recent interest in studying autism and ADHD traits simultaneously in infant sibling studies (i. e., Miller et al., 2018a, 2018b; Shephard et al., 2019; Konke et al., 2022). Autism and ADHD has been characterized by temperament atypicalities. In infancy, low levels of approach, less smiling and high perceptual sensitivity have been associated with later autism traits (del Rosario et al., 2014; Garon et al., 2016) along with low levels of soothability, cuddliness and attention shifting (Clifford et al., 2013; Garon, et al., 2009, Jones et al., 2014; Macari et al., 2017). ADHD traits have been associated with short duration of orienting and poor sustained attention (Auerbach et al., 2008), anger/irritability (Karalunas et al., 2014), extreme levels of positive affect and high activity level in infancy and toddlerhood (Auerbach et al., 2008; Martel et al., 2014; Miller et al., 2016; Miller et al., 2018). Taken together, these prospective studies suggest that there are both shared and specific temperament traits linked to later emerging autism and ADHD traits. To address the question if inherited liability for autism and ADHD impacts infant development, we examined whether proband autism and ADHD traits were associated to the younger siblings’ temperament traits during infancy.

Both from a theoretical and a clinical point of view, it is important to understand how variation in traits are linked to familial effects, versus more idiosyncratic gene-by-environment (G × E) effects within an individual (Manuck & Mccaffery, 2014). In the case of autism, ADHD and most temperament traits, shared familial influences are likely to consist to a large extent by additive genetic effects. We intend to extend recent studies by examining the potential links between proband autism and ADHD traits and temperament traits in the younger sibling, and secondly if these associations are specific or shared. Given the co-occurrence of the two conditions and that temperament patterns present differently in autism and ADHD, we hypothesized that proband traits would be associated to both specific and shared temperament traits in the infant siblings. Presumably similar temperament traits as have been found in longitudinal studies were expected, e.g., negative emotionality in form of higher fear in relation to proband autism and increased anger in relation to proband ADHD traits, whereas higher levels of surgent behaviors, i.e., higher activity level, would be associated with proband ADHD traits.

## Method

### Participants

Participants were recruited to two ongoing longitudinal studies: The British Autism Study of Infant Siblings (BASIS) and the Early Autism ADHD Sweden (EASE). Both studies follow the development of infants with an older sibling with autism from early infancy to school age. The sample for the present study consisted of 216 infants (*n* = 115 from BASIS and *n* = 101 from EASE) with a family history of autism before autistic traits emerge (*M* = 9.39 months, *SD* = 1.03; for participant characteristics, see Table 1), and an older sibling diagnosed with autism (*M* = 7.06 years, *SD* = 2.63).

**Table 1:** Participant characteristics (mean/SD) of infant siblings and probands

	*N*	*M (SD)*	Range	Skewness (SE)	Kurtosis (SE)
Infant measures					
Age (months)	216	9.39 (1.03)	7–12		
Sex (girls:boys)	104:112				
Approach	212	5.46 (0.93)	2.8–7	− 0.58 (0.17)	− 0.26 (0.33)
Vocalization	212	3.41 (0.80)	1.4–6.9	− 0.21 (0.17)	− 0.22 (0.33)
High Intensity Pleasure	212	4.62 (0.64)	2–7	− 1.24 (0.17)	− 0.44 (0.33)
Smiling	212	4.54 (1.10)	1.1–7	− 0.34 (0.17)	− 0.09 (0.33)
Activity Level	212	4.29 (0.96)	2–6.8	0.03 (0.17)	− 0.49 (0.33)
Perceptual Sensitivity	208	3.54 (1.36)	1–6.6	0.03 (0.17)	− 0.83 (0.36)
Distress of Limitations	212	4.17 (0.94)	1.6–6.8	0.15 (0.17)	− 0.05 (0.33)
Fear	212	2.83 (1.19)	1–6.9	0.68 (0.17)	0.31 (0.33)
Falling Reactivity	212	5.07 (1.05)	1.2–6.9	− 0.88 (0.17)	0.72 (0.33)
Sadness	211	3.72 (1.01)	1–6.8	− 0.06 (0.17)	− 0.09 (0.33)
Low Intensity Pleasure	212	4.75 (1.11)	1.7–7	− 0.12 (0.17)	− 0.57 (0.33)
Cuddliness	212	5.54 (0.88)	1.7–7	− 1.33 (0.17)	2.63 (0.33)
Duration of Orienting	212	2.85 (1.17)	1–6.3	0.69 (0.17)	0.12 (0.33)
Soothability	210	5.35 (0.91)	1.7–6.8	− 0.78 (0.17)	1.33 (0.34)
MSEL Composite	211	103 (14)	66–139	− 0.04 (0.17)	− 0.46 (0.33)
*Proband measures*					
Proband Sex (girls:boys)	28:156				
SCQ Age (years)	184	7.06 (2.63)	4–16		
SCQ Total	184	21.03 (8.07)	0–38	− 0.15 (0.18)	− 0.51 (0.36)
CRS-3 Age (years)	138	8.81 (2.63)	6–19		
CRS-3 Total	137	16.88 (7.13)	0–30	− 0.18 (0.18)	− 0.64 (0.41)

*MSEL* mullen scales of early learning composite score; *SCQ* social communication questionnaire; *CRS-3* conners rating scale; *SD* standard deviation; *SE* standard error

Clinical diagnostic assessments of the older sibling were evaluated as part of recruitment. All infants were born at term (at least 32 weeks of gestation), and at the point of recruitment had no known medical or developmental condition.

### Measures

#### Infant Temperament

The Infant Behavior Questionnaire Revised (IBQ-R; Gartstein & Rothbart, 2003) was used to assess infant temperament. The IBQ-R is a well-established parent report measure of temperament in infants (< 12 months of age) and comprises 3 broad factors (negative affectivity, surgency and orienting/regulation) with several subscales for each factor (only the subscales were used in the current study). Surgency includes approach, vocalization, high intensity pleasure, smiling and activity level. Negative affectivity comprises distress to limitations, fear, falling of reactivity and sadness. Finally, orienting/regulation comprises low intensity pleasure, cuddliness, duration of orienting and soothability. Parents rate the frequency of behaviors for specific situations during the last week on a 7-point scale (never to always, or “does not apply”). The standard IBQ-R, with 191 items organized into 14 subscales (Gartstein & Rothbart, 2003), and IBQ-R short version based on 97 items organized into 14 subscales (Putnam et al., 2014), were used. The standard IBQ-R was used in the data collection between year 2011–2014 (*n* = 140 from BASIS) and the short version of the IBQ-R was implemented in the data collection 2014 (*n* = 100 from EASE) and used onwards. With regard to convergent and predictive validity, the shorter version is comparable to the standard IBQ-R scales (Putnam et al., 2014). Mean scores on respective factor were used as independent variables. Cronbach’s alpha for all temperament traits fell within the good to excellent reliability range with α ≥ 0.80 (Taber, 2018), except for sadness (α = 0.76, and distress of limitations (α = 0.78), considered as acceptable level of internal consistency.

#### Autistic Traits

The Social Communication Questionnaire (Rutter, Bailey, & Lord, 2003) was used to examine proband autism traits. The SCQ is a parent-report questionnaire designed to screen for autism traits in children from 4 years and with a mental age over 2 years. The SCQ consists of 40 “yes”/”no” questions on communication skills and social functioning and is based on the Autism Diagnostic Interview (ADI-R; (Lord et al., 1994). The SCQ has established validity with the ADI-R and a diagnosis of autism (Berument et al., 1999).

#### ADHD Traits

The Conner’s Rating Scale- 3 (CRS-3; Conners, 1989, 2008) was used to assess ADHD traits in the older siblings. The CRS-3 is based on parent ratings of ADHD and related problem behaviors in children and adolescents. Parents rate behavior that has been challenging over the preceding month using a four-point likert-scale at levels of appropriateness (e.g., “Not true at all” = 0), and frequency (e.g., “Very frequent” = 3). Scales include oppositional behaviors, cognitive problems, inattention, hyperactivity, anxious-shy, perfectionism, social problems, psychosomatic, a global index, DSM-IV symptom subscales, and an ADHD Index. The CRS-3 shows good psychometric properties and is a valid questionnaire for detecting ADHD traits (Conners, 2008).

#### Developmental Level

The Mullen Scales of Early Learning (MSEL; Mullen, 1995) is a standardized, behavioral measure used to assess the infant’s developmental level in five domains: gross and fine motor, visual reception, receptive language, and expressive language. The early learning composite is a standard score (*M* = 100; *SD* = 15) for overall cognitive ability. The scores from MSEL was used for participant characteristics and added as a covariate in the regressions. The MSEL shows good convergent validity with respect to nonverbal and verbal IQ (Bishop et al., 2011).

### Procedure

Parents completed the IBQ-R when the infant sibling was between 7 and 12 months of age together with the MSEL, and other measures (not included in this study) as part of the two large longitudinal sibling-design studies (BASIS and EASE). Parents completed the CRS-3 and the SCQ when the older sibling had reached at least 5 years of age (CRS-3) and 4 years of age (SCQ).

### Ethical Considerations

Informed consent was obtained from all participating parents included in the study. All procedures performed were in accordance with the ethical standards of the institutional and/or national research committee and with the 1964 Helsinki declaration and its later amendments or comparable ethical standards. The study was approved by the National Ethics Committee in Stockholm, Sweden and by the National Research Ethics Service (London, UK).

### Statistical Analysis

Temperament and probands’ symptom data were converted to z-scores and thereafter screened for outliers (> 3 SD). No univariate outliers were detected. Assumptions of multivariate normality were evaluated using Cook’s distances for the whole sample with all variables. These analyses revealed two extreme multivariate outliers (Cook’s D > 1) that were removed, but this did not change the pattern of result. Analysis of normality revealed that some measures (see Table 1) showed moderate to low skewness and kurtosis, and in order to correct for this non-normal distribution, bootstrapped statistical tests were used in all following analyses (Field, 2013). We used Pearson’s correlations between the predictor variables (probands’ autism and ADHD traits) and outcome variables (infant temperament traits) to examine the correlations before conducting further analyses. To reduce the false discovery rate (FDR), we applied the Benjamini Hochberg (BH) correction to each group of pair-wise comparisons (Benjamini & Hochberg, 1995). The BH critical value was set to 0.05. The correlations surviving BH FDR corrections were retained for regression analyses. To answer the question if traits of autism and/or ADHD in the older proband were specifically or commonly related to temperament in the infant siblings, multiple regression analyses were conducted for each significant temperament trait as dependent variables (DV), and autism and ADHD traits as simultaneous independent variables (IV) in the models, controlling for their overlap. Proband and infant age and sex, infant developmental level and study site were added as covariates in the regression models.

## Results

### Preliminary Analyses

Sample characteristics and descriptive statistics for all study variables are presented in Table 1. Proband data contained 24.1% missing data for CRS-3 (ADHD-traits) and 22% missing data for SCQ (autism traits), missing data for the other variables were below 5%. Multiple imputation (MI) estimation was used to account for missing data. Little’s MCAR test indicated that data were missing completely at random for the included variables. We conducted concurrent correlations between predictors (proband autism and ADHD traits) and potential control variables (proband age and sex). Probands’ autism and ADHD traits correlated significantly (*r* = 0.31, *p* < 0.001), which was also true for autism traits and gender (*r* = 0.29, *p* < 0.001), with higher levels of autism traits for boys. There were no sex differences in the infant temperament traits (all *p*´s > 0.05).

We found several differences related to site, both in the predictors and the dependent variables. There was a significant difference in age of the infant siblings between the two sites, *t*(214) = −  17.66, *p* < 0.001, with younger infant siblings in the BASIS sample (*M* = 8.64, *SD* = 0.81) compared to the EASE sample (*M* = 10.23, *SD* = 0.43). Developmental level (as measured by the MSEL Composite) also differed, with slightly higher levels, *t*(214) = 3.47, *p* = 0.04, for the younger infant siblings in BASIS (*M* = 106, *SD* = 15) than in EASE (*M* = 100, *SD* = 12). The infant siblings´ temperament ratings were significantly higher in the BASIS (*M* = 4.55, *SD* = 0.89) than in EASE (*M* = 3.98, *SD* = 0.94) for activity level, *t*(214) = 4.55, *p* < 0.001. The same trend was found in the ratings for site and fear, *t*(214) = 2.74, *p* = 0.007, with significantly higher fear-ratings in the BASIS (*M* = 3.01, *SD* = 1.11) than in the EASE (*M* = 2.58, *SD* = 1.23). Finally, parent-ratings of infant soothability was significantly lower in the BASIS (*M* = 5.72, *SD* = 0.89) than in the EASE (*M* = 5.03, *SD* = 0.78) cohort, *t*(214) = − 6.02, *p* < 0.001.

Probands’ age and sex did not differ across the sites. However, autism traits were significantly higher, *t*(214) = 6.18, *p* = 0.001, for BASIS (*M* = 23.57, *SD* = 6.63) than for EASE (*M* = 17.56, *SD* = 7.65). Similarly, ADHD-traits were significantly higher, *t*(214) = 6.18, *p* = 0.001, for BASIS (*M* = 23.57, *SD* = 6.63) than for probands in the EASE cohort (*M* = 17.56, *SD* = 7.65).

### Zero-Order Correlations Between Predictors and Temperament Traits

Pearson´s correlations between the predictors (proband autism and ADHD traits) and the infant temperament traits are summarized in Table 2.

**Table 2:** Correlations (Pearson’s r) between infant temperament traits and autism and ADHD traits in probands

Factor	Proband traits	
Temperament traits	Autism	ADHD
Surgency		
Approach	− .24^***,a^	− .02
Vocalization	− .008	− .04
High intensity pleasure	− .07	− .03
Smiling	− .009	.14^*^
Activity	.16^*^	.22^***,a^
Perceptual sensitivity	− .03	− .13
Negative affect		
Distress to limitation	.04	.13
Fear	.26^***,a^	.05
Falling reactivity	− .19^**,a^	− .12
Sadness	.13	.20^*^
Orienting/Regulation		
Low intensity pleasure	− .01	.01
Cuddliness	− .17^**,a^	− .06
Duration of orienting	− .09	.02
Soothability	− .29^***,a^	− .14^*^

*p < .05, **p < .01, ***p < .001

^a^Significant correlation with correction for multiple testing by using Benjamini Hochberg FDR

Only the correlations that were significant at the FDR adjusted p-values were used in the following regressions. Higher levels of probands´ autism traits correlated with lower levels of infant approach, *r*(184) = 0.24, *p* = 0.001, lower soothability, *r*(216) = − 0.30, *p* < 0.001, higher levels of fear, *r*(216) = 0.25, *p* = 0.002, lower levels of cuddliness, *r*(216) = − 0.17, p = 0.01 and falling reactivity, *r*(216) = − 0.19, *p* = 0.005. Higher proband autism traits were correlated with higher activity level in the infant sibling, *r*(216) = 0.16, *p* = 0.02, but this correlation did not hold after FDR corrections.

In relating proband ADHD traits and the younger siblings’ temperament, we found that higher levels of proband ADHD traits correlated with higher levels of infant activity level, *r*(216) = 0.23, *p* = 0.002. Lower rates of smiling, *r*(216) = .− 14, *p* = 0.04 and lower levels of infant soothability, *r*(216) = 0.14, *p* = 0.03 were not significant after FDR corrections.

### Linear Regression Models Between Proband Symptoms (Autism and ADHD) and Infant Temperament

In the next step, we conducted separate bootstrapped linear regression analyses with proband autism and ADHD traits as simultaneous predictors and the significant temperament traits as dependent variables. Proband and infant age and sex, infant MSEL composite score (ELC) and study site were added as covariates in all models. We kept with our original data analysis plan to examine the full sample, but when site was significant in the regression models we conducted separate regressions split by study site. Regression analyses with respective temperament trait for the full sample are presented in Table 3.

**Table 3:** Linear model of predictors of temperament traits, with 95% bias corrected and accelerated confidence intervals (CI) based on 1500 bootstrap samples

	Beta^a^	95% CI lower	95% CI upper	*p* value
Activity model (*n* = 184)				
Proband SCQ	− 0.05	− 0.03	0.02	0.18
Proband CRS	0.18	0.01	0.06	0.01 +
Proband age	− 0.11	− 0.09	0.07	− 0.12
Proband sex	− 0.11	− 0.76	0.12	− 0.12
Infant age	0.09	− 0.11	0.27	0.41
Infant sex	− 0.04	− 0.32	0.18	− 0.61
Infant ELC	0.10	− 0.01	0.02	0.18
Site	− 0.26	− 0.81	− 0.19	0.001 +
Fear model (*n* = 184)				
Proband SCQ	0.17	− 0.001	0.05	0.06
Proband CRS	0.05	− 0.02	0.05	0.54
Proband age	− 0.05	− 0.08	0.05	0.54
Proband sex	0.04	− 0.31	0.63	0.57
Infant age	− 0.09	− 0.14	0.33	0.39
Infant sex	0.17	0.06	0.70	0.02 +
Infant ELC	0.06	− 0.008	0.02	0.40
Site	− 0.21	0.05	− 0.98	0.07
Approach model (*n* = 184)				
Proband SCQ	− 0.25	− 0.05	− 0.01	0.005 +
Proband CRS	0.02	− 0.02	0.03	0.77
Proband age	0.05	− 0.03	0.06	0.53
Proband sex	− 0.04	− 0.51	0.28	0.57
Infant age	0.09	− 0.11	0.27	0.41
Infant sex	− 0.10	− 0.44	0.07	0.16
Infant ELC	0.21	0.003	0.02	0.005 +
Site	0.01	− 0.41	0.46	0.94
Soothability model (*n* = 184)				
Proband SCQ	− 0.16	− 0.04	− 0.01	0.05
Proband CRS	− 0.01	− 0.03	0.02	0.85
Proband age	− 0.01	− 0.05	0.05	0.96
Proband sex	− 0.07	− 0.56	0.17	0.34
Infant age	0.09	− 0.14	0.29	0.39
Infant sex	− 0.03	− 0.32	0.20	0.68
Infant ELC	0.04	− 0.006	0.01	0.58
Site	0.24	0.04	0.88	0.03 +
Falling reactivity model (*n* = 184)				
Proband SCQ	− 0.02	− 0.02	0.02	0.79
Proband CRS	− 0.07	− 0.04	0.02	0.36
Proband age	− 0.11	− 0.10	0.02	0.16
Proband sex	− 0.18	0.17	0.80	0.02 +
Infant age	− 0.18	− 0.44	0.07	0.11
Infant sex	− 0.01	− 0.30	0.28	0.92
Infant ELC	0.05	− 0.01	0.02	0.55
Site	0.28	0.10	1.06	0.02 +
Cuddliness model (*n* = 184)				
Proband SCQ	0.15	− 0.03	0.001	0.09
Proband CRS	− 0.01	− 0.03	0.02	0.86
Proband age	0.12	− 0.009	0.09	0.13
Proband sex	0.05	− 0.19	0.42	0.53
Infant age	− 0.04	− 0.24	0.17	0.73
Infant sex	− 0.04	− 0.31	0.16	0.61
Infant ELC	− 0.02	− 0.01	0.01	0.75
Site	0.09	− 0.25	0.59	0.47

^+^significant after Benjamini Hochberg FDR correction

Multicollinearity was tested with the variance inflation factor (VIF). All predictor and control variables had a VIF < 2.5, indicating low likelihood of multicollinearity.

**Fig. 1:**
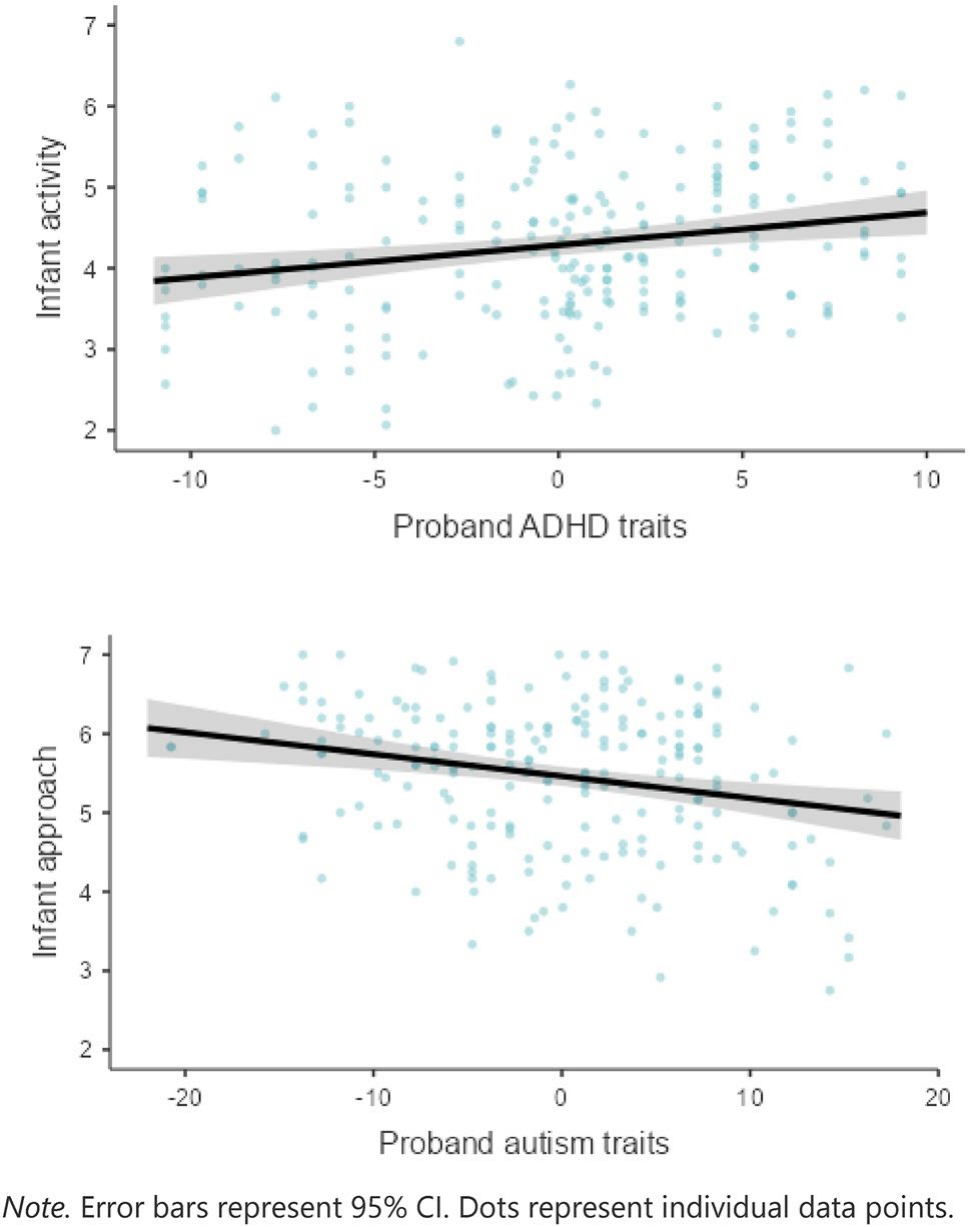
Separate linear regressions across sibling traits with 95% Confidence Intervals (CI)

The first regression model showed that proband autism traits, and infants’ developmental level (as measured by the MSEL composite score), were associated with lower infant approach, adjusting for proband ADHD traits. Proband and infant age and sex did not affect the association (see Fig. 1).

In a separate regression, higher proband autism traits were only marginally associated with increased infant levels of fear and further, sex of the infant was associated with infant fear. To explore these sex differences, we ran separate regressions for boys and girls which indicated a specific association in boys between proband autism traits and infant levels of fear (*β* = 0.25, SE = 0.01, *p* = 0.04), but this relation was not significant in infant girls. Proband ADHD traits were associated with infant activity levels, with higher levels of proband ADHD traits related to higher infant activity (see Fig. 1). Site and probands’ age also contributed to infant activity, with younger probands being associated to higher activity levels in the infant siblings. To explore potential site-differences, we ran separate regressions for the two sites which confirmed similar associations between proband ADHD traits and infant activity, for BASIS (*β* = 0.22, SE = 0.02, *p* = 0.02), although only marginally for EASE (*β* = 0.20, SE = 0.01, *p* = 0.06).

The regression model with soothability as dependent variable indicated a trending association between proband traits of autism and infant soothability (*β* = 0.16, SE = 0.01, *p* = 0.05), which did not hold for Benjamini Hochberg corrections. Due to associations between site and soothability, we ran separate regressions split by site, which confirmed associations between autism traits and soothability, but in the BASIS cohort only.

## Discussion

The aim of this study was to examine associations between proband traits of autism and ADHD and temperament traits in their younger infant sibling. Given that autism and ADHD run in families, we investigated if proband traits could be informative of infant behaviors which could be of developmental concern. We found that proband autism and ADHD traits, respectively, provided unique information about the infant’s temperament, with proband autism traits associated to lower infant approach, and proband ADHD traits associated to higher infant activity level, also after control for both infant and proband age and sex. Infant developmental level as measured by MSEL composite score was explaining unique variance in the model with infant approach but not in the model with infant activity level. Although the effects were small to modest, we reason that these particular associations across siblings reflect shared familial effects (e.g., additive genetic effects). These findings add to the few, but accumulating, studies investigating how proband traits may provide information about the younger sibling (Girault et al., 2020, 2022; Mous et al., 2017). Importantly, the proband-sibling associations that have been reported in earlier studies are found in domains such as language and adaptive behaviour (Girault el al., 2020), proposed as endophenotypes, rather than autism traits or diagnosis. This emphasize the need to focus on familial genetic contributions to early behavioural traits that precedes autism but aggregates in families rather than on aggregation of the core features of the condition. Our study adds to this literature by (1) using proband traits of both autism *and* ADHD based on the high number of children with co-occurring conditions, and (2) exploring common and specific associations to the younger sibling’s temperament traits. This enables us to measure potential liabilities at a very early age before core characteristics of these conditions emerge, which is a key feature in order to better understand etiology and for identifying possible targets for early identification and support.

In terms of temperament associations, we found that higher levels of proband autism traits was specifically associated with low approach behavior in the infants, a result that withstood control for proband ADHD traits and demographic covariates. This finding aligns with prior research reporting lower levels of approach in infants with elevated likelihood of autism compared to typically developing infants (Clifford et al., 2013). Infant developmental level, as measured by the MSEL, explained unique variance in infant approach. One possible explanation for this association is that infants who are high in approach may be more likely to seek out new experiences and opportunities for learning, which can facilitate their cognitive development. Infants who are low in approach are characterized as being more cautious and reserved in new situations which could result in less learning opportunities. Indeed, extreme levels of either high or low approach could be reasons for concern. This finding needs further investigation but highlights the need to consider developmental level when examining infant approach behaviours.

We also found that proband autism was associated with infant soothabillity, meaning difficulties in regulation. This finding was not significant after BH correction. Similarly, higher levels of fear were marginally related to proband autism traits, but not to ADHD traits. A recent study looking at longitudinal associations found a similar pattern, that infant fear was associated with later autism traits and co-occurring anxiety, but not with later ADHD (Shephard et al., 2019), suggesting distinct pathways to autism and ADHD early in life. Our result adds to these findings by showing that similar associations apply across siblings, although not significant in our study.

Proband ADHD traits, but not autism traits, predicted higher infant activity level. This finding is consistent with previous reports linking early activity level to later ADHD symptoms in typically developing children (Frick et al., 2019), as well as in infants with a family history of autism (Miller et al., 2018a, 2018b; Shephard et al., 2019), and a family history of ADHD (Sullivan et al., 2015). Such findings imply that early temperamental activity may be specifically related to ADHD rather than autism both in relation to levels of ADHD traits in early and mid-childhood ADHD as shown previously (Frick et al., 2019; Shephard et al., 2019) and to levels of familial ADHD traits as found in our sample. The possibility exists that activity level during infancy may be overlapping with the core features of ADHD (Kostyrka-Allchorne et al., 2020). Nevertheless, it is important to note that the association between infant activity level and later ADHD symptoms was only modest, consistent with the findings of (Kostyrka-Allchorne et al., 2020), who reported a modest but significant correlation between parent-reported infant activity level and later ADHD symptoms. Specifically, activity level measured in later infancy (> 13 months) was more strongly associated with childhood ADHD than in earlier infancy, which may reflect parents' increased awareness of their child's activity as they become more mobile. Indeed, this may explain why we found only modest associations between activity level and ADHD traits. Certainly, it should be noted that our study assessed associations between traits across siblings rather than within individuals, which may have implications for the strength of our findings. It is still unclear whether infant temperamental activity represents an endophenotype that contributes to ADHD development, or whether it is a manifestation of hyperactivity itself. Still, given its potential involvement in ADHD, temperamental activity warrants careful consideration as a potential target for quantitative genetic studies i.e., utilizing polygenic risk scores.

Except for infant activity level, proband ADHD traits did not specifically relate to any other aspect of temperament in the younger sibling. These few findings may depend on the young age of the infants, suggesting that temperamental vulnerabilities in relation to ADHD may develop later as compared to autism for which core features emerge earlier in development. We also reasoned that the associations with autistic traits may be stronger due to difference in variance between trait levels in the probands, which could impact the associations with the infant siblings´ temperament scores. However, this was not true since we found slightly more variance in autistic traits than in ADHD traits. Indeed, the modest associations may also be explained by other factors that may modulate the link between temperament traits, such factors may be both internal as well as external. Infants as young as 10 months as in our sample are closely bound to their caregiver(s) why external factors, such as parenting may be an important moderating factor to take into consideration.

Finally, we found that proband traits of autism, but not ADHD, made unique contributions to the explained variance in level of infant soothability. Studies of within-individual associations have found lower levels of soothability in toddlers with autism and other developmental delays as compared to typically developing children (Macari et al., 2017; Miller et al., 2016). However, the association was only found in one of the sites (BASIS). This might depend on the age differences of the infants across the sites, with infants in the BASIS being slightly younger than in the EASE, although adding age as a covariate in the analyses did not support this assumption. Another plausible reason might be that parenting, and consequently parent’s ratings of their children, differs across cultures. A general trend was that the parent ratings were slightly higher (e.g., proband traits of autism and ADHD, activity level), or lower (e.g., soothability) in BASIS pointing at *more* difficulties or concerns.

### Strengths, Limitations, and Future Directions

By using parent-reported measures of both infant temperament and proband traits of autism and ADHD our measures potentially include rater-biases resulting in inflated correlations. However, although the rater was the same in the majority of the cases (mothers reported more frequently than fathers), the evaluation of behaviors were done for different children (infant sibling and proband), at different ages and at different time-points which is known to reduce such biases (Nigg, 2006). Although parent-ratings are commonly used in these kinds of studies and are well validated, ideally, they should be accompanied by comparisons with children with typical likelihood for these conditions, objective measures, and longitudinal follow-up data. Regarding our results in relation to ADHD traits in the probands, we found specific relations only between infant temperamental activity and the ADHD phenotype across siblings. While our study only found modest associations between activity level and ADHD traits across siblings, previous research indicate that activity level plays an important role in the development of ADHD (Kostyrka-Allchorne et al., 2020; Nigg, 2006). A recent meta-analysis found that higher activity levels in infants and toddlers predicted later ADHD traits and diagnosis, even after controlling for other aspects of temperament such as negative emotionality and effortful control (Joseph et al., 2023). Our findings align with recent research demonstrating a consistent predictive relationship between high activity level in infants and later ADHD. Together, this provide evidence for the notion that activity level may serve as an endophenotype in ADHD, representing an intermediate trait between genetic influences and the manifestation of ADHD traits. Finally, further research is needed to examine whether the observed associations across siblings, specifically in relation to approach and activity level in our study, may represent inherited developmental vulnerabilities and contribute to the developmental substructure of autism and ADHD (Constantino et al., 2021). Additionally, it is important to investigate the potential cascading effects of these temperament traits during development and their impact on functioning across multiple domains (Constantino, 2021; Masten & Cicchetti, 2010).

## Conclusion

We used an across sibling approach to examine familial autism and ADHD traits in relation to early emerging temperamental traits in infant siblings. By using this design, we found that proband autism traits were associated with lower levels of approach in the infant siblings and that proband ADHD traits were associated with higher activity levels in the infant siblings. These findings suggest that inherited liability for autism and ADHD may influence temperament already in infancy. By comparing associations obtained within person over time with associations obtained between relatives, we may better understand the developmental processes that leads to later autism and/or ADHD traits.
